# Supplementary material for: The Frequency of Meal-Replacement Products Drinking and All-Cause, CVD, and Cancer Mortality
Source: Nutrients. 2024 Nov 2;16(21):3770. doi: 10.3390/nu16213770 (PMC11547669; doi:10.3390/nu16213770)
Supplement: Supplementary file 1 [file nutrients-16-03770-s001.zip › nutrients-3249821-supplementary.pdf]

The Frequency of Meal-Replacement Products Drinking and All-cause, CVD and Cancer Mortality

SUPPLEMENTARY MATERIALS

Figure S1

Tables S1-S2

Figure S1. The flowchart of inclusion criteria

Table S1. The scores assigned to each frequency level when calculating P values for trend associations

Table S2. Sensitivity analyses for MR drinking with all-cause, CVD and cancer mortality

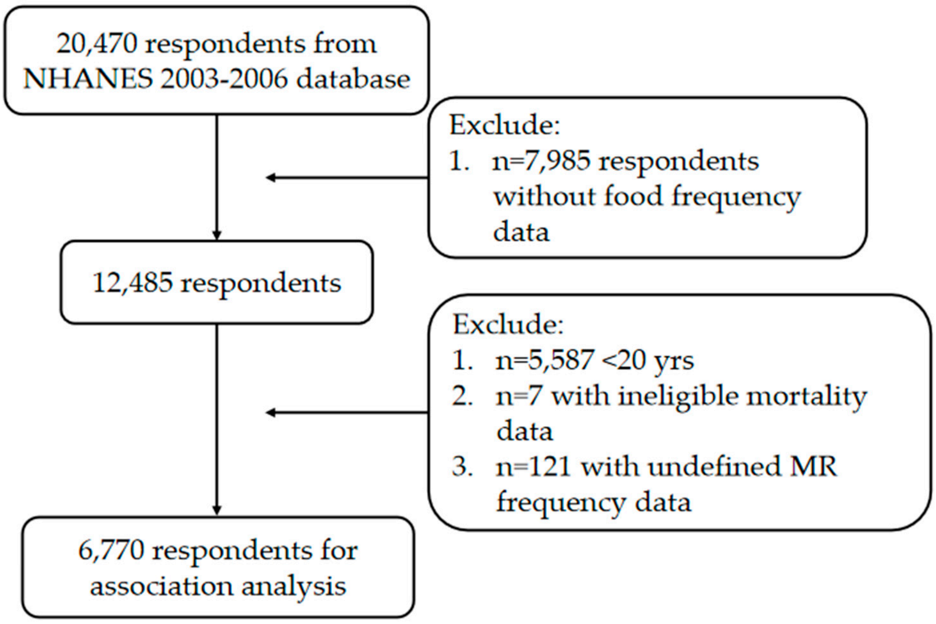

Figure S1. The flowchart of inclusion criteria

Table S1. The scores assigned to each frequency level when calculating P values for trend associations

Frequency Levels	Assigned Scores
Never	0
1 times per month or less	0.03
2-3 times per month	0.08
1-2 times per week	0.21
3-4 times per week	0.5
5-6 times per week	0.79
1 time per day	1
2-3 times per day	2.5
4-5 times per day	4.5
6 or more times per day	7

Table S2. Sensitivity analyses for MR drinking with all-cause, CVD and cancer mortality

Sensitivity analysis	Groups	All cause		CVD		Cancer	
		HR (95%CI)[1]	*P*	HR (95%CI)[1]	*P*	HR (95%CI)[1]	*P*
Multiple imputation[2]	Seldom	1.00		1.00		1.00	
	Monthly	1.21(0.99-1.47)	0.062	1.13(0.73-1.76)	0.596	1.39(0.87-2.24)	0.178
	Weekly	1.48(1.15-1.90)	0.006	1.39(0.93-2.08)	0.114	1.22(0.71-2.08)	0.474
	Daily	1.60(1.26-2.04)	<0.001	1.74(1.15-2.64)	0.012	0.82(0.39-1.73)	0.605
Combine the weekly and daily group	Seldom	1.00		1.00		1.00	
	Monthly	1.26(1.01-1.58)	0.039	1.11(0.74-1.65)	0.609	1.49(0.94-2.35)	0.088
	Combined[3]	1.53(1.29-1.81)	<0.001	1.50(1.11-2.03)	0.008	1.14(0.75-1.72)	0.548
Exclude deaths within the first two years	Seldom	1.00		1.00		1.00	
	Monthly	1.16(0.91-1.48)	0.229	0.99(0.64-1.54)	0.969	1.24(0.73-2.09)	0.426
	Weekly	1.43(1.13-1.82)	0.003	1.29(0.83-2.00)	0.252	1.11(0.63-1.95)	0.712
	Daily	1.47(1.10-1.96)	0.008	1.88(1.19-2.97)	0.007	0.52(0.19-1.43)	0.205
Exclude CVD cases at baseline	Seldom			1.00			
	Monthly			0.79(0.44-1.42)	0.437		
	Weekly			0.97(0.54-1.73)	0.915		
	Daily			1.55(0.85-2.80)	0.151		
Exclude cancer cases at baseline	Seldom					1.00	
	Monthly					1.39(0.80-2.39)	0.241
	Weekly					1.14(0.62-2.10)	0.677
	Daily					0.96(0.42-2.22)	0.93
Exclude diabetes cases and respondents who were in diabetic diets or other kinds of special diets at baseline	Seldom	1.00		1.00		1.00	

	Monthly	1.55(1.20-1.99)	0.001	1.29(0.81-2.06)	0.291	1.72(1.05-2.81)	0.030
	Weekly	1.54(1.20-1.96)	0.001	1.20(0.74-1.92)	0.460	1.12(0.64-1.98)	0.685
	Daily	1.37(1.00-1.87)	0.048	1.32(0.76-2.30)	0.320	0.70(0.30-1.65)	0.414
Keep respondents who had weight lose or weight retain intentions	Seldom	1.00		1.00		1.00	
	Monthly	1.08(0.72-1.62)	0.709	1.11(0.56-2.20)	0.770	1.18(0.51-2.70)	0.704
	Weekly	1.50(1.07-2.12)	0.020	1.15(0.59-2.24)	0.675	0.79(0.32-1.95)	0.604
	Daily	1.48(0.77-2.84)	0.237	2.97(1.16-7.64)	0.024	0.75(0.10-5.49)	0.773
Exclude respondents with implausible energy intake records[4]	Seldom	1.00		1.00		1.00	
	Monthly	1.23(0.97-1.55)	0.082	1.12(0.75-1.68)	0.587	1.40(0.86-2.26)	0.174
	Weekly	1.51(1.21-1.89)	<0.001	1.22(0.80-1.86)	0.349	1.28(0.77-2.13)	0.343
	Daily	1.48(1.14-1.92)	0.004	1.70(1.10-2.61)	0.016	0.67(0.30-1.54)	0.351
Exclude respondents who reported being unable to eat because of problems with teeth, mouth or dentures	Seldom	1.00		1.00		1.00	
	Monthly	1.24(0.98-1.56)	0.075	1.06(0.70-1.62)	0.779	1.54(0.96-2.46)	0.072
	Weekly	1.45(1.15-1.83)	0.002	1.25(0.93-1.93)	0.323	1.26(0.74-2.12)	0.396
	Daily	1.47(1.12-1.94)	0.006	1.73(1.15-2.72)	0.017	0.99(0.48-2.05)	0.976
Exclude respondents with abnormal low levels of serum albumin or missing serum albumin values[5]	Seldom	1.00		1.00		1.00	
	Monthly	1.22(0.96-1.54)	0.106	1.03(0.67-1.59)	0.898	1.33(0.81-2.17)	0.264
	Weekly	1.49(1.17-1.89)	0.001	1.43(0.94-2.19)	0.096	1.07(0.61-1.88)	0.805
	Daily	1.52(1.15-2.01)	0.003	1.63(1.02-2.60)	0.043	0.95(0.44-2.04)	0.900

[1]All the sensitivity analyses were multi-variate adjusted including age, sex, race, educational levels, occupation, family income, BMI, physical activity, smoking, drinking,

disease histories (CVD, cancer, diabetes, hypertension, COPD, hyperlipidemia), and HEI as covariates, except for analyses excluding baseline cases (When excluding baseline cases, the corresponding disease was not included in the covariates).

[2]R package "mice" was used for multiple imputation. Proportional odds models were used and all covariates with Nelson-Aalen estimator of cumulative hazard were included.

[3]The combined group included the 'weekly' and 'daily' group.

[4]Implausible energy intake was defined as: men <=800 kcal/day or >=4000 kcal/day; women <=500 kcal/day or >= 3,500 kcal/day.

[5]abnormal low levels of serum albumin was defined as: serum albumin<35g/L.
